# Editorial: Emergent Behavior in Animal-Inspired Robotics

**DOI:** 10.3389/fnbot.2022.861831

**Published:** 2022-03-22

**Authors:** Alex Gomez-Marin, Yisi Zhang

**Affiliations:** ^1^Behavior of Organisms Laboratory, Instituto de Neurociencias (CSIC-UMH), Alicante, Spain; ^2^Princeton Neuroscience Institute, Princeton University, Princeton, NJ, United States

**Keywords:** animal, robot, emergent behavior, biology, machine, scientist, reductionism

The *ménage à trois* between animals, scientists, and robots offers an interesting intersection of *Umwelts*, namely, meaningful environments that are different but partially overlap. While engineers build machines to solve our human problems, biologists may do so to better grasp how their laboratory creatures solve their own. Recalling the line of Wordsworth's poem, when it comes to animals, “[w]e murder to dissect.” However, instead of taking animals apart, one can appreciate the elusive nature of behavior by putting robots together into functioning wholes. Drawing on the similarities (and differences) between animals and “animats” that land within human knowledge thus far offers a methodological engine to expand the scientist's world into the directions of ethology and robotics (see Figure 1).

**Figure 1:**
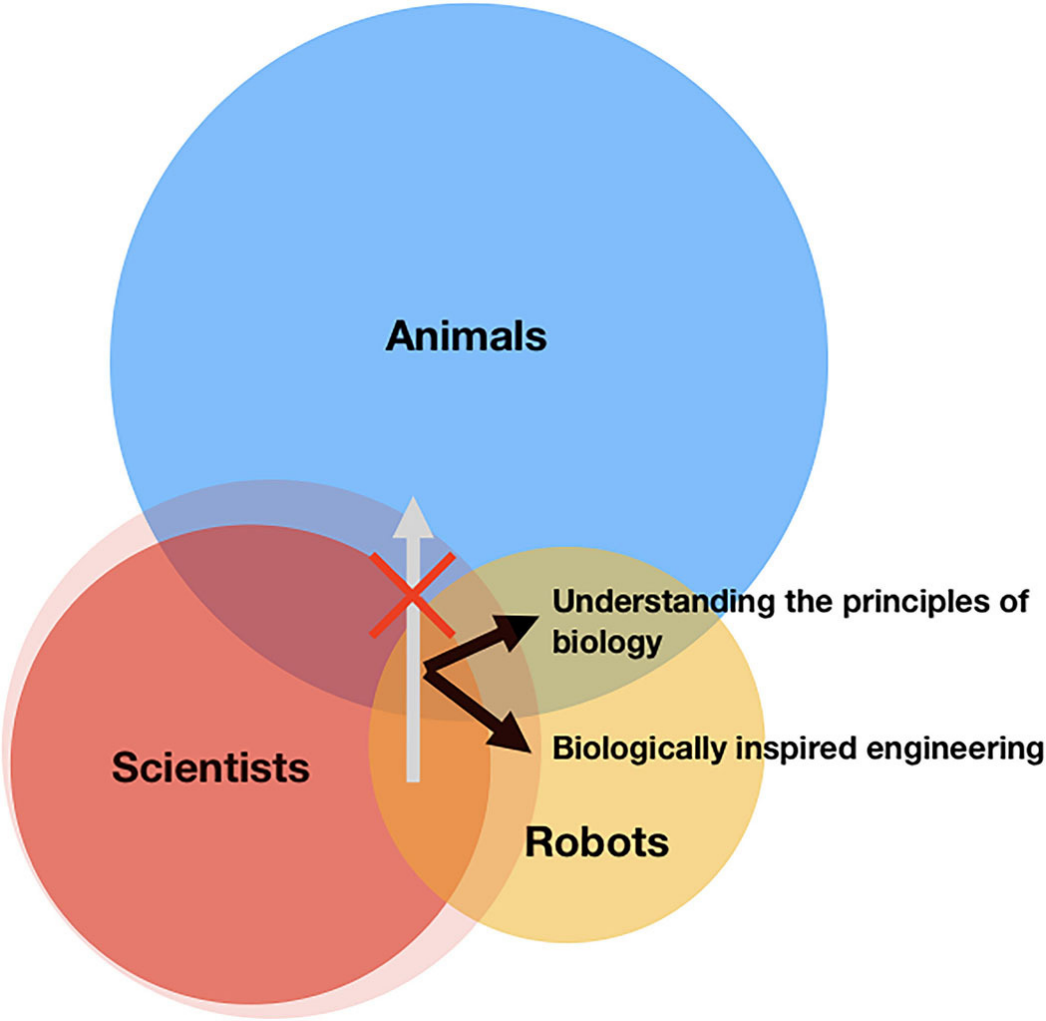
Human, animal, and robot worlds. Fruitful avenues and fallacious ones, in the use of machines as living organisms. The red region stands for the limit of scientists' knowledge, the blue region the world of animal behavior, and the yellow region the scope of robotics. The intersection between red and blue represents the current understanding of animal behavior; between red and yellow is the progress in robotics; between blue and yellow means the similarities between animal and machine. The intersection at the center is the mechanistic understanding of animal behavior that can be justifiably applied to robotics and vice versa (e.g., biocybernetics). The black arrows indicate how the intersection at the center can direct the development of robotics and understanding animal behavior, and the gray one identifies an erroneous application of robotics to studying life.

In this exchange, artificial systems can help us understand living ones. Animals produce and maintain themselves, grow and develop, while robots are extrinsically built. But both are made of interactive materials and may share organizing principles that allow them to accomplish things in the real world. By conceiving “animats as animals,” we can test our intuitions as to the validity of the conceptual and methodological approaches we routinely use to study neuro-ethological questions. For instance, we can design a robot solution, pretend we forgot it, and then compare our answers when studying robot and animal behavior in homologous situations. The approach then provides improved educated guesses in situations where, of course, we cannot know the solution *a priori*. The robot becomes a serious (and more ethical) “toy model” for animal research.

Building machines having animals in mind has further benefits: we can test ideas in the real world rather than in the idealized realms of numerical simulations or the disembodied conceptions of neural processing. Robot behavior faces the same physical constraints as actual animals; our whiteboard models and computer codes often do not. When it comes to behavior, animals and machines (well, their engineers) have “skin in the game.” Physical instantiations of biological ideas in the form of machine models naturally embrace and face real-world friction, complex shapes, soft material, error, wear, and imperfection. Robotic implementations can thus serve to simulate hypotheses sometimes unfeasible to test in biological systems themselves (as we have access to the parameters of the system) or in numerical models of those systems (if we do not, those parameters are not mere idealizations). In robots, the rules that constrain the emergence of behavior manifest with greater realism.

In this Research Topic, entitled “*Emergent Behavior in Animal-Inspired Robotics*,” we present several studies demonstrating how robotics is a powerful construct in helping us understand complex sensorimotor behaviors, learning, collective behaviors, and even evolution. These studies, encompassing social interaction, vocal production, and goal-directed reaching, apply numerical and physical models to simulate natural lives and test hypotheses in biology. Specifically, Reséndiz-Benhumea et al. test the social brain hypothesis with a minimal model of social interaction and neural network. Amador and Mindlin establish a low-dimensional biomechanical model to simulate the production of complex birdsongs. He and Ogmen illustrate self-organized goal-directed reaching *via* a feedback loop. Matić et al. show how features of biological movement can be naturally achieved by implementing a hierarchical architecture based on perceptual control. These studies follow a general practice that integrates biologically-based building blocks to explore the emergence of behavior under controllable conditions. They illustrate how animal-inspired robotics, as a methodology, keeps both prompting the robotics field and generating insights into animal behavior (as illustrated in Figure 1).

Such a machine-based expansion of our understanding of living organisms has its limits. Although it goes without saying, one must indeed make explicit that the regime of biology that is formalizable by the robotic framework is not the entire province of the study of life. While animal-inspired robotics is a handy methodological move, machine-inspired biology can become an “epistemic oxymoron.” Fallacies abound when one seeks to stubbornly infer or deduce biological principles using blind mechanistic assumptions (the forbidden direction in Figure 1). Captured by the image of nature as a grand machine, to insist that living organisms are just current or future machines we have not invented yet simply kicks an entrenched “meta-metaphor” forward; if the “animal as animat” claim is not false, then it is just empty. The map, present or future, is not the territory.

Paradoxically, while roboticists may conceive of their creations as life-like, when it comes to understanding living organisms biologists prefer to treat their creatures as life-less. Reducing their subjects of study into parts, some scientists believe they can get an objective handle on them (and they do, but at what cost? What is lost?). Roboticists, on the contrary, seek to animate their creations. A curious historical example of such a tendency for “sleight of terms” lies in cybernetics. We speak of machines as if they had a purpose (which is only our purpose embodied in their operations), while simultaneously claiming that we, humans, really do not. Here is another example. In the beginning, there were animals, and then we started building machines in their image (not the reverse). But then we convinced ourselves and others that it is more appropriate to say that horses are like trains than that trains are like horses. The same inversion took place in analogies between brains and computers: first computers were built in analogy with neural function, but later we ended up believing that brains are actually computers. We surreptitiously switch terms over and over.

In sum, animals are not machines. Rather, if anything, it is machines that are *like* animals. As heirs of Descartes' fruitful mistake, we are still pondering the cross-roads offered by Darwin's realization: either we embrace that nature is like us (*cogitans*, thinking, feeling, alive!) or we claim that we are like what we thought nature was (*extensa*, mindless, dull, mechanic). The second choice is depressing, and probably also wrong. The etymology of the word robot is “forced labor;” a robot is a slave. The root of the word animal stems from “having breath,” namely, imbued with force and life. Shall we confer to robots what we end up negating to animals? The challenge of animal-inspired robotics and machine-inspired biology is to proceed in a way that generates insights both for the study of animals and machines without conflating apples with iPhones.

## Author Contributions

All authors listed have made a substantial, direct, and intellectual contribution to the work and approved it for publication.

## Conflict of Interest

The authors declare that the research was conducted in the absence of any commercial or financial relationships that could be construed as a potential conflict of interest.

## Publisher's Note

All claims expressed in this article are solely those of the authors and do not necessarily represent those of their affiliated organizations, or those of the publisher, the editors and the reviewers. Any product that may be evaluated in this article, or claim that may be made by its manufacturer, is not guaranteed or endorsed by the publisher.

